# Ophthalmology

**Published:** 1898-05

**Authors:** Alvin A. Hubbell

**Affiliations:** Buffalo, N. Y., Professor of ophthalmology in Niagara University Medical College


### Progress in Medical Science.

### OPHTHALMOLOGY.

Conducted by ALVIN A. HUBBELL, M. D., Buffalo, N. Y.,
Professor of ophthalmology in Niagara University Medical College.

### HEMIANOPIA, WITH ESPECIAL REFERENCE TO ITS TRANSIENT VARIETIES.

DR. WILFRED HARRIS (*Brain*), Autumn number, 1897, discusses quite at length hemianopia and arrives at the following conclusions:

(1) That hemianopia, rarely binasal, more commonly lateral and left-sided, with accompanying constriction of the remaining half fields, may occur as a temporary phenomenon in hysteria.

(2) That hemianopia, due to a vascular lesion of the cuneus, of sudden onset, may commence with marked loss of sight, sometimes amounting to complete amaurosis, and due probably to inhibition of the remaining half-vision center.

(3) That the cortical half-vision centers are not subdivided into centers for light, form and color respectively, and that hemiachromatopsia may be due to a lesion anywhere in the visual path between the chiasma and the cortex.

(4) That quadrantic hemianopia, though strongly suggestive of a cortical lesion, may sometimes be due to a lesion in the internal capsule.

(5) That the macular region of the retina is invariably supplied with nerve fibers on the same plan as the rest of the retina, *i. e.*, each side of it from the corresponding side of the brain. That in all cases of absolute transient hemianopia the dividing line between the seeing and the blind halves invariably passes through the fixation point.

(6) That the cortical center for the macular region in each cuneus is less liable to complete destruction and recovers earlier than the rest of the half vision center.

(7) That cases of persistent hemianopia in which the dividing line passes to one side of the fixation point, leaving it in the seeing half, are to be accounted for, either (*a*) by the escape or partial recovery of the cortical center for the macula, or (*b*) by the acquirement by education of a new fixation point in the retina.

(8) That hemianopic visual spectra of low elaboration, such as red and green lights, or the varieties of scintillating scotoma in migraine, are caused by a discharge in the half-vision center in the cuneus.

(9) That complex visual phenomena of hemianopic type, such as faces, animals, etc., are elaborated in a still higher visual center, which possibly is the angular gyrus; their occurrence in the half field only being due to reflex irritation from a lesion generally in or near the cuneus, but which may be in the optic radiations or optic tract.

(10) That double hemianopia does not necessarily cause permanent amaurosis, in many cases the return of a small area of central vision indicating the escape or recovery of the cortical center for the macula in the cuneus on each side.

(11) That the hemianopia in migraine is due to an epileptic discharge in the half-vision center of one side.

(12) That in many cases an epileptic discharge may originate in or near the half-vision center on one side, in some cases proceeding no further, beyond producing temporary hemianopia, in others producing a typical epileptic fit, and again in others giving rise to unilateral convulsions without loss of consciousness.

(13) That transient hemianopia in such attacks may last for twenty-four hours or longer, and may be due to vascular softening adjacent to, but not involving, the visual center or path.

(14) That transient hemianopia is rare in ordinary Jacksonian epilepsy, and is not liable to occur unless the half-vision center be (1) already slightly damaged, or (2) hypersensitive and prone to spontaneous discharge, as in migraine.

(15) That such transient hemianopia not infrequently accompanies unilateral convulsions in general paralysis, and may possibly occur in uremia.

(16) That the auditory center may be similarly paralysed through spread of the epileptic discharge.

---

### TREATMENT OF BLEPHARITIS MARGINALIS.

Dr. Ayres (*Cincinnati Lancet-Clinic*, October 23, 1897,) believes that in the treatment of blepharitis marginalis, the best results are obtained with a solution of hydrogen dioxide and water, equal parts. This accomplishes the desired result and does not pain the eye. It is to be applied with a bit of absorbent cotton, dipped into the dioxide solution and rubbed along the lashes. This should be kept up until the specific oxidising effect is seen on the scales or crusts, as will be evidenced by the bubbles. The edges of the crusts will begin to separate. They are then to be dried with absorbent cotton.

There is a great advantage in using this remedy in children; it greatly lessens the pain of the treatment. It is also of special value where ointments of all kinds produce more or less irritation and sometimes cause an aggravation of the symptoms.

---

### METHOD OF PRESERVING THE EYE FOR SECTIONING OR DEMONSTRATION.

James R. Slonaker, Ph. D., (*Journal of Applied Microscopy*,) describes the following method of preserving the eye for sectioning or for demonstrating the area of acute vision:

In order that the eye may be well preserved, it must be placed, as soon as possible after death, in Perenyi's fluid: 10 per cent. nitric acid, 4 parts; 95 per cent. alcohol, 3 parts; 0.5 per cent. chromic acid, 3 parts.

The eye should be carefully oriented before removing from the head, by sewing a tag to the outer layers of the sclerotic, so that the orientation may afterwards be exact. In removing the ball it should not be punctured, for this invariably causes wrinkling of the retina. As much as possible of the fat and muscle should be removed before immersing it in the Perenyi. The bulk of the preserving fluid should be ten or fifteen times the size of the eye.

The time that the Perenyi should be allowed to act depends on the size of the eye and the nature of the sclerotic. Small eyes, such as rat, sparrow, etc., are usually left in twenty-four hours and large ones, such as cow, horse, or those in which the sclerotic contains bony plates, thirty-six to forty-eight hours are required. A good deal of latitude may be taken, however, regarding the time.

After the action of Perenyi's fluid, the eye is carried up through the following grades of alcohol, leaving it twenty-four hours in each: 70 per cent., 80 per cent., 90 per cent., 95 per cent., 100 per cent., and, finally, a mixture of equal parts of absolute alcohol and ether. It is then well hardened and ready for celloidin imbedding. Before putting into celloidin a window is cut in the same plane as the desired sections, exposing the hardened vitreous humor. After this is carefully removed, without injury to the remaining structures, the hollow ball is put into celloidin. He has used three grades of celloidin, ranging from very thin to very thick and has left the eye at least forty-eight hours in each. It can remain longer with better results. It is then mounted on a block in the usual manner and cut in 80 per cent. alcohol.

If one desires only to demonstrate the fovea and not to make sections, the front half of the eye may be cut off after passing through the 80 per cent., but preferably after 95 per cent. alcohol. When thus removed, together with the vitreous humor, the retina is seen spreading smoothly over the posterior half of the ball and the fovea, if present, and the optic papilla may be easily seen. Such demonstration material can be kept permanently in 80 per cent. alcohol.

This method applies only in a gross study and cannot be used successfully for the minute structures of the retinal elements. If such is desired, the Golgi method, as used by Ramon y Cajal, or the "methyl blue" method will admirably serve the purpose.

